# Reinforced Feedback in Virtual Environment for Plantar Flexor Poststroke Spasticity Reduction and Gait Function Improvement

**DOI:** 10.1155/2019/6295263

**Published:** 2019-12-25

**Authors:** Carlos Luque-Moreno, Fátima Cano-Bravo, Pawel Kiper, Ignacio Solís-Marcos, Jose A. Moral-Munoz, Michela Agostini, Ángel Oliva-Pascual-Vaca, Andrea Turolla

**Affiliations:** ^1^San Camillo IRCCS, Via Alberoni, 70, 30126 Lido di Venezia, Italy; ^2^Departamento de Enfermería y Fisioterapia, Universidad de Cádiz, Av. Ana de Viya, 52, 11009 Cádiz, Spain; ^3^Hospital Universitario Virgen Del Rocío, Av. Manuel Siurot S/n, 41013 Sevilla, Spain; ^4^Department of Behavioural Sciences and Learning, Linköping University, 58183 Linköping, Sweden; ^5^Departamento de Fisioterapia, Universidad de Sevilla, C/ Avenzoar, 6, 41009 Sevilla, Spain

## Abstract

**Background:**

Ankle spasticity is a frequent phenomenon that limits functionality in poststroke patients.

**Objectives:**

Our aim was to determine if there was decreased spasticity in the ankle plantar flex (PF) muscles in the plegic lower extremity (LE) and improvement of gait function in stroke patients after traditional rehabilitation (TR) in combination with virtual reality with reinforced feedback, which is termed “reinforced feedback virtual environment” (RFVE).

**Methods:**

The evaluation, before and after treatment, of 10 hemiparetic patients was performed using the Modified Ashworth Scale (MAS), Functional Ambulatory Category (FAC), and Functional Independence Measure (FIM). The intervention consisted of 1 hour/day of TR plus 1 hour/day of RFVE (5 days/week for 3 weeks; 15 sessions in total).

**Results:**

The MAS and FAC reached statistical significance (*P* < 0.05). The changes in the FIM did not reach statistical significance (*P*=0.066). The analysis between the ischemic and haemorrhagic patients showed significant differences in favour of the haemorrhagic group in the FIM scale. A significant correlation between the FAC and the months after the stroke was established (*P*=−0.711). Indeed, patients who most increased their score on the FAC at the end of treatment were those who started the treatment earliest after stroke.

**Conclusions:**

The combined treatment of TR and RFVE showed encouraging results regarding the reduction of spasticity and improvement of gait function. An early commencement of the treatment seems to be ideal, and future research should increase the sample size and assessment tools.

## 1. Introduction

Stroke patients suffer several deficits that affect (mildly to severely) the cognitive, psychological, or motor areas of the brain, at the expense of their quality of life [1]. Although rehabilitation techniques do not only act on the motor deficits [2], the effects associated with the interruptions of the corticospinal tract, as well as the subsequent adaptive changes, commonly require specific interventions. Among them, the most important changes are muscle weakness, loss of dexterity, cocontraction, and increased tone and abnormal postures [3].

Hemiparesis is the most common problem in poststroke patients, and its severity correlates with the functional capabilities of the individual [4], being that impairment of gait function is one of the most important limitations. Furthermore, weakness of the ankle muscles caused by injury to supraspinal centres and spasticity are the most frequent phenomena that limit functionality [5]. The degree of spasticity of the affected ankle plantar flex (PF) muscles primarily influences gait asymmetry [6], which is, in addition to depression, another independent factor for predicting falls in ambulatory stroke patients [7]. Physiological changes in the paretic muscles, passive or active restraint of agonist activation, and abnormal muscle activation patterns (coactivation of the opposing lower extremity (LE)) have been shown to occur after a stroke and can lead to joint stiffness (foot deformities are present in 30% of stroke patients) [8], deficits in postural stabilization, and reduced muscle force generation [9]. To enhance this postural stability during gait, it seems that poststroke patients with impaired balance and paretic ankle muscle weakness use a compensation strategy of increased ankle muscle coactivation on the paretic side [10].

Scientific evidence shows that the use of mixed techniques with different physiotherapy approaches under very broad classifications (i.e., neurophysiological, motor learning, and orthopaedic) provides significantly better results regarding recovery of autonomy, postural control, and recovery of LE in the hemiparetic patient (HP) as compared to no treatment or the use of placebo [11]. Within the latter techniques, we may emphasize the relearning of motor-oriented tasks [12], as well as other approaches based on new technologies (e.g., treadmill [13], robotics [14–16], and functional electrical stimulation (FES) [17]), which are often used as additional treatments to traditional rehabilitation (TR). However, some of these emerging therapies, such as vibratory platforms [18], have not been shown yet to produce as positive results as the prior ones. Thus, obtaining better results with mixed and more intensive rehabilitation treatment has been demonstrated [19, 20]. Therefore, we propose to add the use of virtual reality (VR) techniques to TR to optimize results. We can use the label “VR-based therapy” because it acknowledges the VR system as the tool being used by the clinician in therapy, not as the therapy itself. It is essential to transfer the obtained gains in VR-based therapy to better functioning in the real world [21]. In this way, the intersection of a promising technological tool with the skills of confident and competent clinicians will more likely yield high-quality evidence and enhanced outcomes for physical rehabilitation patients [22].

The application of VR to motor recovery of the hemiparetic LE (HLE) has been addressed by several authors in the last decade [23–28], obtaining satisfactory results, in general terms, in the increase of walking speed [22, 24, 25], cortical reorganization, balance, and kinetic-kinematic parameters. Other authors have reported improvements in the balance of patients treated with nonimmersive VR systems based on video games, using specific software and with the guidance of a therapist [29]. A recent study showed that VR-based eccentric training using a slow velocity is effective for improving LE muscle activity to the gastrocnemius muscle and balance in stroke [30]; however, the spasticity of PF muscles was not analysed in any of these studies.

Virtual reality acts as an augmented environment where feedback can be delivered in the form of enhanced information about knowledge of results and knowledge of performance (KP) [31]. There are systems that use this KP through the representation of trajectories during the execution of the movement, as well as visualizing these once performed, to visually check the amount of deviation from the path proposed by the physiotherapist. Several studies demonstrated that this treatment enriched by reinforced feedback in a virtual environment (RFVE) may be more effective than TR to improve the motor function of the upper limb after stroke [31, 32]. In our study, the use of a VR-based system, together with a motion capture tool, allowed us to modify the artificial environment with which the patient could interact, exploiting some mechanisms of motor learning [33, 34], thus allowing greater flexibility and effective improvement in task learning. This system has been highly successful in the functional recovery of the hemiparetic upper extremity [31, 33–36], but its combined effect with TR on the LE has not yet reported conclusive data [37]. The continuous supply of feedback during voluntary movement makes it possible to continuously adjust contractile activity [38], thus mitigating increments in spasticity and cocontraction processes of the patient. These settings are of great significance in motor control, and certain variables (such as the speed of the movement) can be controlled, having a direct influence on spasticity. In this line, the aim of this study is to determine if there is a decrease in the spasticity of the PF muscles and improved gait function, following a program that includes the combination of TR and VR with reinforced feedback, which is called “reinforced feedback virtual environment” (RFVE).

Moreover, as a complementary aim, we analysed the modulatory effects of demographic and clinical factors on the recovery of patients treated with TR and VR. The analysis of the influence of these modulatory variables was focused on better highlighting what type of patients would benefit most from the combined treatment of TR and VR. Particularly, we looked into the effects of age and time elapsed from the moment the stroke occurs until the patient starts neurorehabilitation. As shown in various studies, a better outcome for treatment can be expected for younger patients and for those who start the treatment earlier [39]. Also, comparisons were made between patients with an ischemic and haemorrhagic stroke, since differences in their recovery prognostic have been reported elsewhere, with better outcomes for the latter group [40].

## 2. Materials and Methods

### 2.1. Sample

In the present study, the sample consisted of 10 male poststroke patients (5 with right hemisphere injury and 5 with left hemispheric injury; 6 with ischemic strokes and 4 with haemorrhagic strokes). The demographic and clinical characteristics of both groups of patients are presented in Table 1. Human experimentation was approved by the Ethical Committee of the University of Seville (Spain). Each patient provided written consent allowing the use of their demographic and clinical information for research purposes. Any personal information that could identify them was removed to preserve their anonymity.

**Figure 1:**
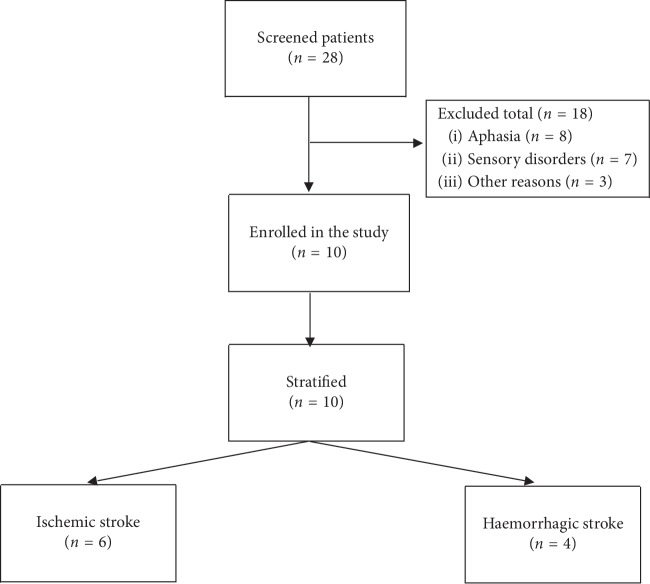
Flowchart of participants through the study.

The inclusion criteria were as follows: patients with a single stroke (ischemic or haemorrhagic) included in a physiotherapy program (1 hour/day, 5 days/week) and never treated before with RFVE. The exclusion criteria were as follows: evidence of cognitive impairment (patients underwent a cognitive screening before inclusion in the study), De Renzi test score of below 62 or receptive aphasia that would alter the understanding of tasks, and conducting additional rehabilitation with other technologies that could influence the results (e.g., robotics, FES, and vibration platforms) (Figure 1).

### 2.2. Assessment and Intervention

A preintervention evaluation, an intervention based on the objectified deficits, and a postintervention evaluation were performed on the 10 patients described above. It is important to highlight that before the date of the first assessment, they all had received TR in the initial period following the stroke.

#### 2.2.1. Assessment of Spasticity

- Spasticity of PF muscles of the hemiparetic ankle was assessed using the Modified Ashworth Scale (MAS), with the patient resting in supine position. The MAS is the most widely used and extended measure to quantify hypertonia of any joint [41, 42], testing the resistance of muscles to fast manual stretching, and providing reliable measurements of spasticity when patients are evaluated by a single examiner [41]. Data obtained with the MAS have been statistically rescaled so that a score of 1 + on the MAS corresponds to a numerical score of 2, 2 corresponds to a numerical score of 3, 3 corresponds to a numerical score of 4, and 4 corresponds to a numerical score of 5 (thus, we consider a numerical category 1 +, which includes such a scale).

#### 2.2.2. Assessment of Functionality

- Functional Ambulatory Category (FAC) [43]: it was designed to examine the levels of assistance required for walking along a 15-meter corridor, without receiving any technical help. It is divided into 6 categories, ranging from 0 (does not walk) to 5 (normal).
- Functional Independence Measure (FIM) [44]: it is a scale constructed from 7 levels of performance. Eighteen items have been defined within 6 performance areas: self-care, sphincter control, mobility, strolling, communication, and social knowledge. The maximum score for each item is 7, and the minimum score for each item is 1. So, the maximum score obtained would be 126, and the minimum score obtained would be 18.

According to the VR intervention, patients underwent 1 hour/day of treatment based on RFVE, in addition to the TR already performed for 1 hour/day, 5 days/week for 3 weeks (a total of 15 sessions). Subsequently, a clinical evaluation (MAS, FAC, and FIM) pre- and post-intervention, comparing the results with specified statistical methods, was performed.

Moreover, the TR session was focused on the overall functional recovery of the patient (including the upper limb) [2, 45–48]. Patients allocated to the TR group received specific rehabilitation of the LE consisting of passive, assisted, and active exercises in many directions in the lower limb workspace (e.g., coxofemoral joint flexion and extension, abduction and adduction, internal and external rotation, knee flexion, and extension) and mixed techniques with different approaches [11]. Exercises were performed in the sitting and standing positions, and each of the training programmes was personalized to the motor capacities of patients. The individual task-oriented exercises were selected for each patient in accordance with their current mobility conditions (e.g., exercises for postural control in the standing or sitting position instead of gait training). Then, the exercise programme was progressively increased in terms of complexity by the physiotherapist in charge of the treatment (e.g., go up and downstairs or exercises to improve dynamic balance), according to results from the functional assessment. Thus, exercises performed by patients in the TR group were addressed to achieve the best functional skills for balance and walking autonomy.

**Figure 2:**
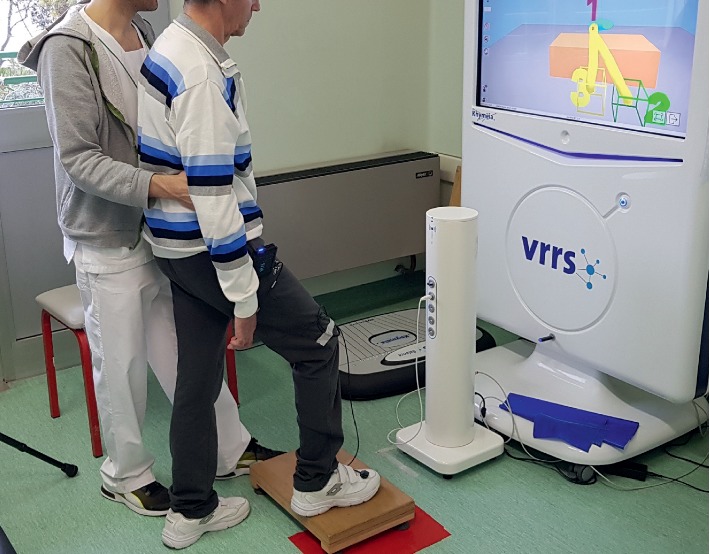
Patient carrying out a task set out by the physiotherapist in front of the RFVE equipment.

The treatment based on RFVE was specifically centred on the recovery of the HLE [28, 43]. The RFVE equipment used consisted of a computer workstation connected to a 3D motion tracking system (Polhemus 3Space FasTrak Vermont, USA) and a high-resolution LCD projector that displayed virtual scenarios on a large screen (Figure 2). The 3D motion tracking system detected the position of the electromagnetic sensor placed on the LE. The flexible software, developed at the Massachusetts Institute of Technology (Cambridge, MA, U.S.), was used to create several motor tasks for the LE. The nature and complexity of the motor tasks were adapted to the previously evaluated deficiencies, gradually increasing the difficulty and providing the variability that boosted motor learning.

**Figure 3:**
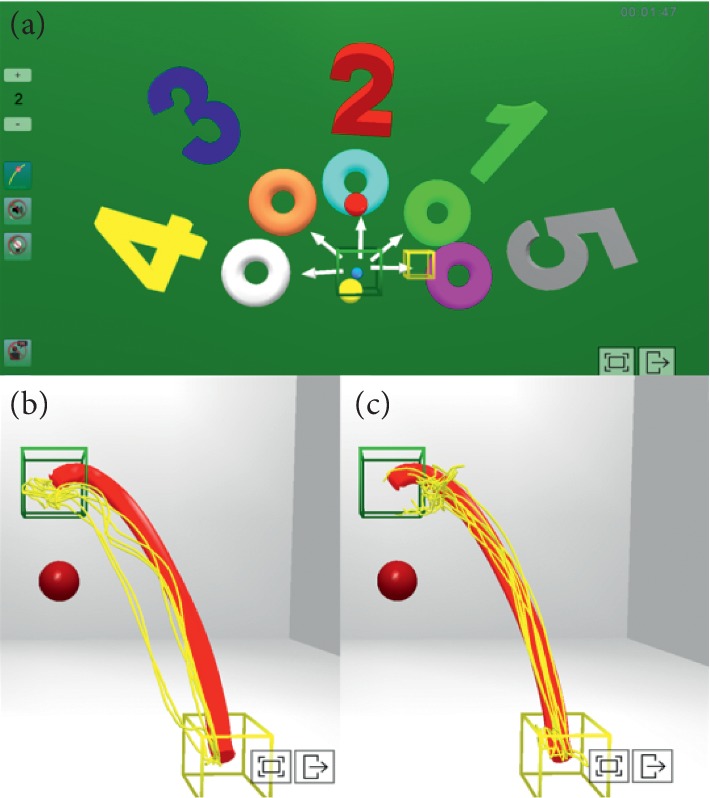
Different trajectories proposed to patients in the virtual scenario. (a) Star of numbers with different directions to follow, moving the foot on the ground without losing contact with the plant. In (b) and (c), the ideal path proposed by the physiotherapist (red) and the different tests performed by the patient (yellow) are shown. As can be seen, the executed trajectories (yellow) approximate the proposed ideal trajectory (red) from pretraining (b) to posttraining (c).

In the virtual scenario, the starting position and the features of the target were determined to facilitate the perception of errors and their correction by the subject (learning by imitation) [37, 49], enabling the acquisition of motor skills [43] and employing artificially reinforced feedback. While performing the task, subjects obtained information on the movement of their limb (KP) through the virtual representation of the trajectory carried out by the sensors (Figure 3). Amplification of the visual and auditory feedback was controlled, providing calculations of the score for each trial of the task and the use of the “virtual teacher” (T). The latter gives the patient continuous guidance on the ideal speed at which the movement should be carried out. It was possible to modulate the rate of the T, controlling spasticity while performing the given task and performing a higher-quality motion [4], as well as the reduction of the ankle muscle coactivation on the paretic side [10]. In line with previous studies focusing on the motor rehabilitation of the upper limb [31], the differences in muscle activation patterns of the LE were considered. Because the motor control mechanisms for both LEs are affected during poststroke gait [5], specific interventions were carried out for their normalization. When the exercises were performed in a standing position, the patient was asked to stand on the nonparetic limb and perform open kinetic trajectories with the paretic limb to improve the oscillation phase. When asked to stand on the paretic limb, a sensor was placed on the dorsal side of the nonparetic foot (this was performed to optimize the proprioception of the paretic side induced by the movement of the centre of pressure on the supporting foot when moving the nonparetic side towards different trajectories in open kinetic chain). This exercise in a closed kinetic chain over the paretic LE could be an effective treatment method to improve gait patterns in stroke patients, since it would provide constant sensory input from the affected foot [50]. Besides, the associated eccentric work of these exercises would have a direct relationship with the reduction of the best activation of the gastrocnemius muscle, as long as it is conducted at the low speed [38], which in our case was controlled by using the T guide [37]. In linear paths, dragging the foot on the ground from the starting point to the arrival point (different numbers), additional feedback (especially the speed of realization through the T) was provided for the realization of the different directions in which the spasticity of the FP musculature (speed-dependent) had to be modulated to perform a correct FD movement. At the same time, it was important to repeat more specific tasks that included the effect of gravity until reaching the point of arrival, providing visual-auditory feedback to achieve better performance until approaching the ideal trajectory. Theoretically, the best movement should be repeated, emulating a reference model as exactly as possible, with the aim to achieve the best motor performance [34]. Afterwards, it was possible to show the patient the performance of the task (Figures 3(b) and 3(c) offered the patient the possibility to visualize, in 3D, the ideal trajectory (red lines) and the different repetitions (yellow lines) to see at what point and in which direction the real trajectory moved further away from the ideal, thus being able to perform a more specific training of specific points of the trajectory) to obtain feedback that could help in its correction (KP). PF weakness is a determinant of kinetic asymmetry during gait in poststroke individuals walking with high levels of effort [51]. For that reason, it was paramount to perform exercises aimed at enhancing muscle strength while avoiding spasticity and cocontraction phenomena by continuously providing feedback to the patient. For example, controlled exercises (in trajectory, distance, and speed) of heel lift in load to improve the maximum peak of plantar flexion during take-off (sensor in the heel) [37].

### 2.3. Statistical Analysis

**Figure 4:**
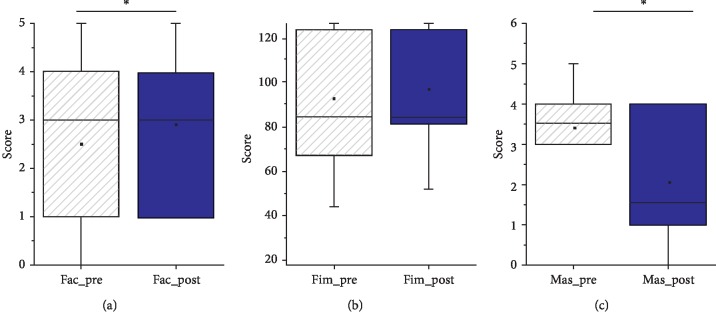
Boxplots showing the group scores pre- and post-treatment on each scale. Means are also represented by a square. Individual scores from each patient have also been included.

Mean and median scores on the different scales for each group are represented in Figure 4. Pre- and post-treatment comparisons were performed by using repeated-measures ANOVAs after having confirmed the parametricity of the different variables.

Additionally, the treatment effects on the ischemic (*n* = 6) and haemorrhagic (*n* = 4) groups were also analysed in light of previous studies reporting differences in their responsiveness to the rehabilitation treatment [40]. Both groups' scores on the different scales pre- and post-treatment were compared by using Mann–Whitney tests. Moreover, *differential variables* (henceforth “*Diff variables*”) were calculated by subtracting the pretreatment scores from the posttreatment scores for each of the scales (i.e., *Diff. FIM*, *Diff. FAC*, and *Diff. MAS*). Both groups were compared on these variables by using Mann–Whitney tests to account for differences in their response to the treatment.

Finally, correlation analyses between the *Diff variables* and the different demographic and clinical variables collected were conducted to account for the modulatory effect of these variables on the effect of the treatment. Nonparametric Spearman analyses were used.

## 3. Results

### 3.1. Pretreatment and Posttreatment Comparisons

Individual and group scores on each scale pre- and post-treatment are represented in Figure 3. The repeated-measures ANOVAs revealed significant changes in the FAC (*F*(1, 9) = 6; *P*=0.03; partial eta-squared = .4) and MAS (*F*(1, 9) = 5,12; *P*=0.04; partial eta-squared = .36) scales. As shown, the mean FAC scores increased from 2.5 (SE = .6) pretreatment to 2.9 (SE = .5) posttreatment, indexing an improvement in ambulation functionality. Conversely, mean MAS scores decreased from 3.4 (SE = .34) pretreatment to 2 (SE = .56) posttreatment, thus indicating reduced spasticity posttreatment. Besides, increases in the overall scores on the FIM scale were observed, although it did not reach the significance level (*F*(1, 9) = 5; *P*=052; partial eta-squared = .36).

### 3.2. Modulatory Effects of Age, Months after the Stroke, and Stroke Aetiology

Correlation analyses showed no linear associations between age and any of the *differential variables* (i.e., *Diff. MAS, Diff. FAC,* and *Diff. FIM*). However, a significant positive correlation was observed between the number of months after the stroke and *Diff. FAC* (rho = −0.71, *P*=0.05). Those patients who started the treatment earlier after the stroke showed a greater recovery in ambulation functionality. Comparisons between ischemic and haemorrhagic patients on the *differential variables* revealed significant differences in *Diff. FIM* (Table 1), with greater values for the latter group indicating more significant improvements in their independence functionality.

## 4. Discussion

Once the analysis was performed, the results could be considered satisfactory. In this way, significant data pointing to an improvement in gait function measured by the FAC was obtained. Similar results were obtained by other authors that included the use of other VR systems applied to the HLE [23–28]. Although the variability of assessment tools used in this work did not allow us to establish full parallelism, in a study conducted by You et al. [24] in which the FAC was included as the assessment tool into the VR system “IREX VR system,” similar results were obtained. In general terms of overall functionality measured by the FIM, despite some improvements being observed in a group, this change did not reach statistical significance. Since the FIM includes 18 categories (of which only 2 are closely related to gait function), we suggest that this is not adequate to measure its evolution.

Despite the well-known effect of botulinum toxin on the reduction of spasticity, no associated positive effects on the functionality of gait were observed in terms of reduction spasticity [52, 53]. Since the treatment intensity, as well as the learning of new motor skills, promotes cortical reorganization [34], it becomes necessary to implement therapies aimed at optimizing the effects of TR. An interesting finding is the improvement of spasticity in the PF muscles of the HP. In that sense, any of the authors mentioned above reported this deficiency with the use of VR systems applied to the HLE. The reason may be the lack of flexibility of the software of some VR systems used in these studies that, despite being optimal for learning motor tasks, are more limited to control some parameters related to the selective control of the motion. In our case, the ability to continuously monitor and change parameters (such as trajectory and speed of execution), which is closely related to spasticity, could influence the positive results; however, it would be necessary to carry out studies that analysed the specific effect of VR. Furthermore, the degree of improvement in spasticity was higher in the acute patients as compared to chronic patients. This may be due to the gradual establishment of the spasticity in stroke patients, increasing from the first months.

Results suggest that the RFVE system can contribute to TR improving ankle spasticity, besides other interventions, and this may contribute to reducing the risk of falls in patients [7]. However, some limitations of this study should be mentioned here. For example, the sample size was quite small (only 10 patients). Nevertheless, this limitation is not specific to this study and is rather common in the literature, as it affects the generalizability of our results. Another limitation is that no control group was used, that is, a group of patients who only underwent regular physiotherapy (without VR). Finally, since the eccentric exercise, when performed at low speeds, significantly improves the function of the gastrocnemius muscle [30], future research should consider comparing the spasticity measurements obtained through the MAS and the muscle activation pattern from EMG in order to account for spasticity improvements associated with the reciprocal innervation pattern.

With regard to other studies, better results were obtained in patients who started the treatment earlier (fewer months poststroke) [39, 54], with a significant improvement in gait function. This finding would reinforce the idea that early neurorehabilitation may potentiate different physiological processes underlying spontaneous recovery of the brain after an injury. Moreover, no modulatory influence of age on the treatment effect was observed, thus contradicting previous work [39]. Probably, a sample of patients with a wider range of ages would have better highlighted the influence of this variable on neurorehabilitation treatment. Last, in line with other studies [39], the results showed greater functional recovery in the haemorrhagic patients as compared to the ischemic patients. In this respect, similar results have been reported by Paolucci et al. [40] in conventional physical neurorehabilitation treatment.

VR, just as robotics, are consolidated tools for the functional rehabilitation of the upper limb poststroke. The use of the LE to promote locomotor relearning is more recent and presents unique challenges under the complex multisegmental mechanics of gait [55]. For this reason, it is crucial to invest efforts in adapting the reinforced feedback systems to the reeducation of the poststroke gait and find synergies between robotics and VR in order to develop more effective systems.

## 5. Conclusions

Although this study does not present evidence on the additional effects of VR and TR, the combined treatment of TR and RFVE showed encouraging results regarding the reduction of spasticity and improvement of gait function. Early commencement of the treatment seems to be ideal, and future research should increase the sample size and evaluation tools as well as provide two comparison groups between TR and VR.

Rehabilitation treatment could be enriched with the use of RFVE systems. Nevertheless, this tool is not a suitable substitute for an expert professional, since clinical experience is essential for effective use of the system. Therefore, physiotherapists are required to select the most appropriate strategies for each patient and the time of the process, executing them by adapting the parameters related to reinforced feedback to enhance motor learning. Future research is needed to determine the specific additional effects of this treatment.

## Figures and Tables

**Table 1:** Median (25^th^—75^th^ percentiles) for age, months after stroke, and scores on the scales before and after the treatment are presented. Calculated *differential variables* are also included. Mann–Whitney's *U* and *Z* values are indicated along with the corresponding *P* values.

Scales	Ischemic (*n* = 6)	Haemorrhagic (*n* = 4)	*U*	Z	*P*
Age	62.47 (55.82–75.3)	63.47 (52.29–72.33)	10	−0.4	0.76
Months after stroke	7.34 (3.98–12.07)	4.6 (3.11–6.74)	8	−0.85	0.39
FAC pre	3.5 (1.5–4.75)	1.5 (0–3)	5	−1.25	0.13
FAC post	3.5 (2.25–4.75)	2 (1–3.25)	7	−1.08	0.27
FIM pre	112 (88–125)	81 (73.8–91.3)	3	−1.93	0.05
FIM post	109 (86.5–125)	67 (61.3–80.8)	5	−1.5	0.13
MAS pre	2.5 (1–4)	1.5 (0.75–2.5)	9.5	−0.57	0.57
MAS post	3.5 (3–4)	3.5 (3–4.25)	8.5	8.5	−0.78
*Diff. FAC*	0 (0–.25)	1 (.25–1)	5	−1.75	0.08
*Diff. FIM*	0 (0–1.5)	11 (0–14)	3.5	−2.05	0.04
*Diff. MAS*	0 (-2.5–.25)	−1.5 (−4.25, to −0.25)	6.5	−1.22	0.22

## Data Availability

The data used to support the findings of this study are available from the corresponding author upon request.

## Abbreviations

PF: Plantar flexor
LE: Lower extremity
TR: Traditional rehabilitation
RFVE: Reinforced feedback virtual environment
MAS: Modified Ashworth Scale
FAC: Functional Ambulatory Category
FIM: Functional Independence Measure
HP: Hemiparetic patients
FES: Functional electrical stimulation
HLE: Hemiparetic lower extremity
VR: Virtual reality.
